# Hemozoin as a Diagnostic Biomarker: A Scoping Review of Next-Generation Malaria Detection Technologies

**DOI:** 10.3390/bios16010048

**Published:** 2026-01-07

**Authors:** Afiat Berbudi, Shafia Khairani, Alexander Kwarteng, Ngozi Mirabel Otuonye

**Affiliations:** 1 Department of Biomedical Sciences, Parasitology Division, Faculty of Medicine, Universitas Padjadjaran, Bandung 40161, Indonesia; 2 Research Center for Care and Control of Infectious Diseases (RC3ID), Universitas Padjadjaran, Bandung 40161, Indonesia; 3 Department of Biomedical Sciences, Cell Biology Division, Faculty of Medicine, Universitas Padjadjaran, Bandung 40161, Indonesia; shafia@unpad.ac.id; 4 Veterinary Medicine Program, Faculty of Medicine, Universitas Padjadjaran, Sumedang 45363, Indonesia; 5 Department of Biochemistry and Biotechnology, Kwame Nkrumah University of Science and Technology, Kumasi, Ghana; akwarteng@knust.edu.gh; 6 Central Research Laboratory, Nigerian Institute of Medical Research, Yaba, Lagos P.O. Box P.M.B. 2013, Nigeria; mnotuonye@gmail.com

**Keywords:** malaria diagnosis, hemozoin analysis, biosensing techniques, magnetics, optical phenomena, point-of-care testing

## Abstract

Accurate malaria diagnosis is essential for effective case management and transmission control; however, the sensitivity, operational requirements, and field applicability of current conventional methods are limited. Hemozoin, an optically and magnetically active crystalline biomarker produced by *Plasmodium* species, offers a reagent-free target for next-generation diagnostics. This scoping review, following PRISMA-ScR and Joanna Briggs Institute guidance, synthesizes recent advances in hemozoin-based detection technologies and maps the current landscape. Twenty-four studies were reviewed, spanning eight major technology classes: magneto-optical platforms, magnetophoretic microdevices, photoacoustic detection, Raman/SERS spectroscopy, optical and hyperspectral imaging, NMR relaxometry, smartphone-based microscopy, and flow cytometry. Magneto-optical systems—including Hz-MOD, Gazelle™, and RMOD—demonstrated the highest operational readiness, with robust specificity but reduced sensitivity at low parasitemia. Photoacoustic Cytophone studies demonstrated promising sensitivity and noninvasive in vivo detection. Raman/SERS platforms achieved sub-100 infected cell/mL analytical sensitivity but remain laboratory-bound. Microfluidic and smartphone-based tools offer emerging, potentially low-cost alternatives. Across modalities, performance varied by parasite stage, with reduced detection of early ring forms. In conclusion, hemozoin-targeted diagnostics represent a rapidly evolving field with multiple viable translational pathways. While magneto-optical devices are closest to field deployment, further clinical validation, improved low-density detection, and standardized comparison across platforms are needed to support future adoption in malaria-endemic settings.

## 1. Introduction

Malaria remains one of the most persistent global health challenges, particularly affecting populations in low- and middle-income countries across sub-Saharan Africa and Southeast Asia. Timely and accurate diagnosis is crucial for malaria control and elimination, as it enables early treatment, reduces complications, and limits the transmission of the disease. However, currently available diagnostic approaches—namely, microscopy, rapid diagnostic tests (RDTs), and molecular assays—have notable limitations. Microscopy is labor-intensive and operator-dependent, with reduced sensitivity at low parasitemia; antigen-based RDTs may fail due to pfhrp2/pfhrp3 gene deletions or low parasite densities; and polymerase chain reaction (PCR), although highly sensitive, is costly and unsuitable for routine field use [1].

Hemozoin, also known as malarial pigment, is a crystalline by-product of hemoglobin digestion by *Plasmodium* parasites and is an attractive, inherently stable biomarker for malaria diagnosis. Its unique biophysical properties—including paramagnetism, birefringence, optical absorbance, and magneto-optical behavior—enable detection using diverse physical and engineering platforms. These properties have motivated a shift toward hemozoin-based diagnostic technologies that overcome several limitations of antigen- and nucleic-acid-based methods [2].

Hemozoin accumulation is strongly stage-dependent across the intraerythrocytic lifecycle of *Plasmodium*. Early ring-stage parasites contain minimal pigment, whereas trophozoite- and schizont-stage parasites accumulate progressively larger quantities of crystalline hemozoin. In *P. falciparum* infections, mature trophozoites and schizonts are largely sequestered within the microvasculature of deep tissues, while ring-stage parasites circulate in peripheral blood. This biological compartmentalization directly influences both the anatomical accessibility of hemozoin and the achievable limits of detection for different diagnostic approaches [3].

Over the past two decades, a wide range of hemozoin-targeting detection methods have been developed, including magneto-optical techniques, magnetoacoustics and photoacoustic platforms, laser-based detection, depolarized light-scattering systems, and microdevice-integrated biosensors. Magneto-optical systems, for example, have demonstrated detection thresholds approaching 10 parasites/µL under controlled laboratory conditions [1]. Photoacoustic-based platforms, meanwhile, leverage the strong optical absorption of hemozoin to achieve highly sensitive detection in both blood samples and engineered microfluidic systems [4]. In addition, field-deployable devices such as the Gazelle™ have been tested in operational settings, showing high specificity but variable sensitivity depending on parasite density and specieScheme 5.

Despite these promising advances, the evidence on diagnostic performance, feasibility, and readiness for field implementation remains fragmented. Many techniques are still in laboratory or prototype stages, and their performance in detecting low-density or asymptomatic infections—conditions common in elimination settings—requires further evaluation. Furthermore, gaps persist regarding cost-effectiveness, operational requirements, species differentiation, and suitability for low- and middle-income country (LMIC) health systems.

Given the rapid expansion of hemozoin-based diagnostic research and the absence of a comprehensive synthesis, this scoping review aims to map available evidence on hemozoin-based malaria detection technologies. Specifically, the review identifies the types of technologies developed, summarizes their diagnostic performance, and highlights implementation challenges and knowledge gaps to guide future research.

**Figure 1:**
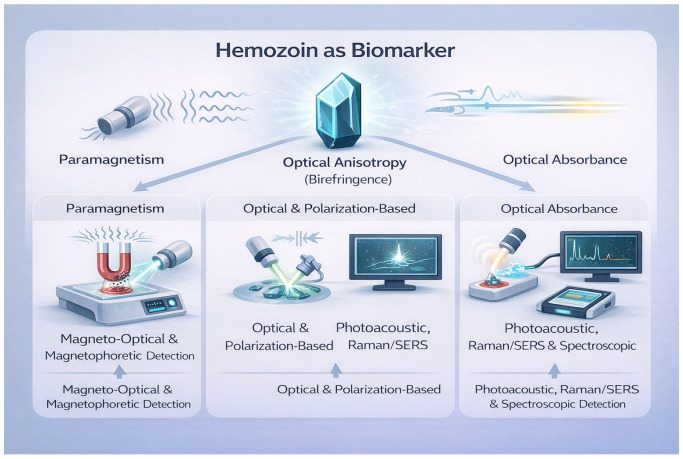
Core biophysical properties of hemozoin and their diagnostic exploitation. This schematic illustrates the three principal physical characteristics of hemozoin—paramagnetism, optical anisotropy (birefringence), and strong optical absorbance—that enable reagent-free malaria detection. Representative diagnostic platforms exploiting each property are shown, including magneto-optical, optical/polarization-based, photoacoustic, and spectroscopic approaches. Polarization symbols are used as visual cues to indicate the exploitation of polarized light and optical anisotropy (birefringence) in the corresponding detection modalities.

To provide an overview of the diagnostic landscape examined in this review, Figure 1 illustrates how the unique biophysical properties of hemozoin—paramagnetism, optical anisotropy, and optical absorbance—directly underpin the diverse diagnostic technologies developed for reagent-free malaria detection. This conceptual map serves as a visual guide for interpreting the diverse detection principles and device platforms described in the subsequent sections.

## 2. Materials and Methods

### 2.1. Review Framework and Protocol

This scoping review was conducted in accordance with the PRISMA-ScR and JBI methodologies. The protocol was developed a priori and prospectively registered on the Open Science Framework. All eligibility criteria, search procedures, and data charting methods were predefined and consistently applied throughout the review.

### 2.2. Information Sources

Two electronic databases were searched: PubMed (MEDLINE) and Scopus (Elsevier). PubMed was selected for its comprehensive coverage of parasitology, malaria diagnostics, and biomedical innovation literature. Scopus was selected in addition to PubMed due to its broad coverage of engineering, optics, applied physics, and interdisciplinary journals relevant to hemozoin-based diagnostic technologies. This scoping review prioritizes translationally relevant diagnostic approaches rather than exhaustive coverage of all engineering developments. Searches were restricted to peer-reviewed primary research published between 2020 and 2025 to capture the most recent advances in hemozoin-based diagnostic development. No restrictions were applied to geographical setting or *Plasmodium* species (clinical samples, experimental models, or in vitro cultures).

### 2.3. Search Strategy

The search strategy was developed iteratively using Medical Subject Headings (MeSH), free-text keywords, and Boolean logic to maximize sensitivity for hemozoin-focused diagnostic studies. Search strings were adapted to each database’s syntax.

- PubMed Search Strategy: (“hemozoin” [MeSH] OR hemozoin OR “malarial pigment”) AND (“malaria diagnosis” [MeSH] OR malaria OR *Plasmodium*) AND (detection OR “diagnostic method” OR biosensor OR magneto-optic OR photoacoustic OR magnetoacoustic OR “light scattering” OR “laser detection”).
- Scopus Search Strategy: TITLE-ABS-KEY (hemozoin OR “malarial pigment”) AND TITLE-ABS-KEY (malaria OR *Plasmodium*) AND TITLE-ABS-KEY (detection OR diagnostic OR magneto-optic OR photoacoustic OR biosensor OR magnetoacoustic OR “light scattering”).

To increase specificity and minimize engineering-only publications, Scopus results were limited to the following subject areas: Medicine; Biochemistry, Genetics and Molecular Biology; Chemistry; Immunology and Microbiology; and Materials Science. No language filters were applied during the search; restriction to English occurred at the screening stage. The search strategy was iteratively developed using MeSH terms, free-text keywords, and input from a malaria diagnostics expert. The final search string was piloted to ensure sensitivity for hemozoin-targeted technologies. Reference lists of included studies were not manually searched due to the highly specialized nature of the topic.

The restriction to studies published between 2020 and 2025 was applied to capture recent technological advances and translational developments in hemozoin-based diagnostics, rather than to provide an exhaustive historical overview.

### 2.4. Eligibility Criteria

Eligibility criteria were defined using the Population Exposure Outcome (PEO) framework and refined to ensure rigorous and reproducible inclusion.

#### 2.4.1. Inclusion Criteria

Studies involving primary research, such as experimental device development, method validation, or cross-sectional diagnostic assessment, were included when they met the following criteria:

- Population: clinical samples, *Plasmodium*-infected blood, or in vitro cultures relevant for diagnostic evaluation.
- Exposure/Concept: evaluation of diagnostic methods that directly detect hemozoin, including but not limited to magneto-optic, photoacoustic, laser-based, depolarized light scattering, magnetic field-assisted, or biosensor-integrated platforms.
- Outcome: reporting at least one diagnostic parameter: sensitivity, specificity, limit of detection (LOD), detection principle, or performance evaluation.
- The search was limited to English-language articles published from 2020 to 2025.

#### 2.4.2. Exclusion Criteria

Studies were excluded if they involved animal models without a diagnostic evaluation component; did not include hemozoin-based detection (e.g., malaria therapy, basic biology); focused solely on physicochemical or mechanistic properties of hemozoin without diagnostic endpoints; were secondary literature (systematic reviews, scoping reviews, narrative reviews), commentaries, editorials, or opinion articles; were non-English or published before 2020.

### 2.5. Selection of Sources of Evidence

All records were imported into Rayyan.ai for automated deduplication and blinded screening. Two reviewers independently screened titles and abstracts using the predefined eligibility criteria. Full texts of potentially relevant studies were retrieved and assessed by the same reviewers. Disagreements were resolved through discussion, and a third reviewer adjudicated when consensus could not be reached. The study selection process is presented in the PRISMA-ScR flow diagram.

### 2.6. Data Charting Process

Data extraction was performed using a standardized charting form, which was piloted on five studies to ensure consistency. Data regarding the following variables were collected: author/year, study design, sample type, detection principle, device/platform, analytical performance metrics (sensitivity, specificity, LOD), sample volume, stage-specific performance, technology readiness level (TRL), and reported limitations. Studies were further categorized according to the primary nature of the evidence provided. Mechanistic and proof-of-principle investigations were included to establish feasibility and signal origin, whereas clinical and field studies were evaluated for diagnostic performance and translational relevance. Mechanistic studies were not interpreted as direct evidence of diagnostic accuracy but as foundational support for the development of hemozoin-based diagnostic approaches.

The TRL assessment followed the 9-level scale adapted for biomedical devices. TRL estimates were assigned based on reported evidence of clinical evaluation, field testing, prototype maturity, and regulatory exposure, following established TRL definitions [5,6]. The ranges shown reflect comparative translational positioning rather than standardized certification. Data extraction was performed independently by two reviewers, and any discrepancies were resolved collaboratively.

### 2.7. Synthesis of Results

A narrative thematic synthesis was undertaken to map the range of technologies and diagnostic approaches. Extracted data were grouped into five predefined analytical domains: magneto-optic techniques; photoacoustic technologies; light-scattering-based detection; magnetic-field-assisted detection; and integrated biosensors and novel platforms. Within each domain, findings were synthesized to examine diagnostic performance, technical advantages, practical limitations, and remaining evidence gaps.

## 3. Results

### 3.1. PRISMA-ScR Flow Summary

**Figure 2:**
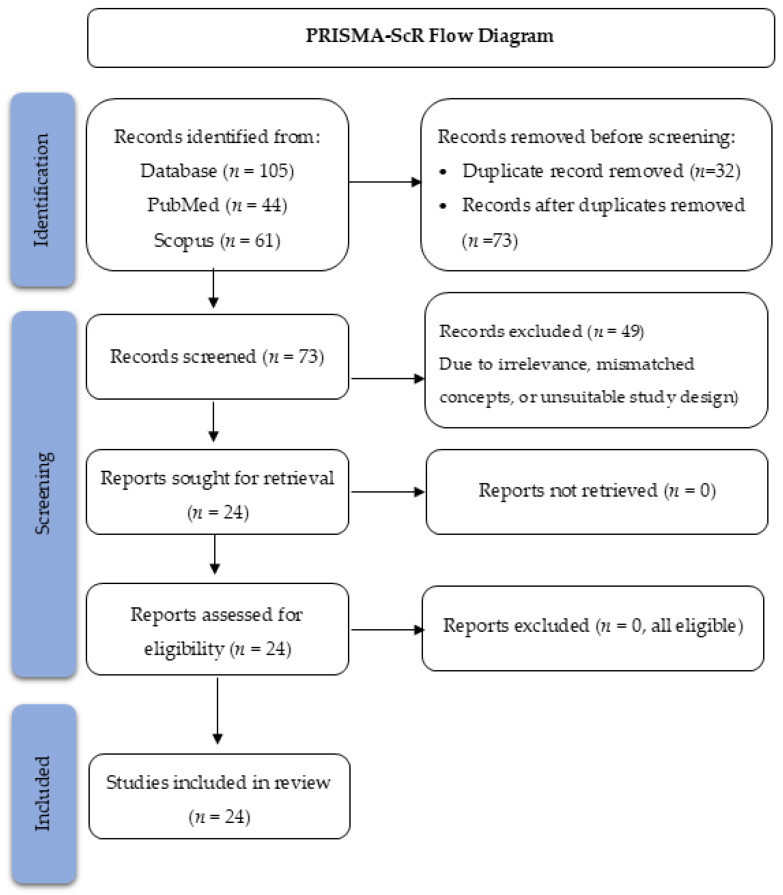
PRISMA-ScR Flow Diagram of Study Selection.

The search identified 105 records (PubMed *n* = 44; Scopus *n* = 61). After removing 32 duplicates, the titles and abstracts of 73 articles were screened. A total of 49 records were excluded due to irrelevance, concept mismatches, or unsuitable study designs. Twenty-four full-text articles were assessed for eligibility, all of which met the inclusion criteria and were included in the final synthesis. The study selection process is summarized in the PRISMA-ScR flow diagram (Figure 2).

The flow diagram illustrates the identification, screening, eligibility assessment, and inclusion process for studies evaluating hemozoin-based malaria diagnostic technologies. A total of 105 records were identified from PubMed (*n* = 44) and Scopus (*n* = 61). After removing duplicates (*n* = 32), 73 records underwent title/abstract screening, of which 49 were excluded due to irrelevance, mismatched concepts, or unsuitable study design. Twenty-four full-text articles were assessed for eligibility, and all were included in the final synthesis (*n* = 24).

### 3.2. Study Characteristics

A total of 24 studies, published between 2020 and 2024, met the inclusion criteria. The studies were conducted across Africa, Asia, Europe, and the Americas, employing a mixture of laboratory analytical designs (*n* = 14), clinical cross-sectional or longitudinal evaluations (*n* = 7), and physicochemical characterisation studies (*n* = 3). Sample materials included *Plasmodium falciparum* cultures, *P. vivax* field samples, β-hematin standards, magnetically enriched erythrocyte fractions, venous whole blood, and in vivo circulating blood interrogated by noninvasive photoacoustics.

Eight major technology classes were identified (Table 1, Supplementary Table S1):

(1) magneto-optical detection (MOD/Magneto-optical Detection & RMOD/Rotating-Field Magneto-Optics) (*n* = 8);
(2) magnetophoretic microdevices (*n* = 1);
(3) photoacoustic detection (*n* = 2);
(4) Raman/SERS spectroscopy (*n* = 3);
(5) optical and hyperspectral imaging (*n* = 3);
(6) NMR relaxometry (*n* = 1);
(7) Smartphone microscopy & pigment-containing leukocytes (*n* = 3)
(8) flow cytometry, chemical, and nonlinear optical methods (*n* = 3).

The studies varied widely in analytical sensitivity, sample volume requirements, detection principles, and readiness for implementation in malaria-endemic settings.

**Table 1:** Technology Groups, Core Detection Principles, Operational Settings, and Key Findings Across 24 Hemozoin-Based Malaria Detection Studies.

Technology Group	Study	Method Summary	Core Principle	Typical Setting	Summary of Findings
Magneto-optical (Gazelle/Hz-MOD)	[7]	Point-of-care MO detection for *P. vivax*	Magnetic alignment of hemozoin → optical modulation	Field/ clinic	High specificity; stable *P. vivax* detection; reduced sensitivity at low parasitemia
	[8]	MO detection of *P. falciparum*	Static magnetic modulation	Hospital	Excellent specificity; weaker detection of low-density ring-stage infections
	[9]	MO detection of *P. knowlesi*	Magnetically induced optical contrast	Hospital	High specificity; first MO evaluation for *P. knowlesi*
	[10]	Multicenter Hz-MOD field evaluation	Optical modulation under a magnetic field	Field/clinic	Strong multi-site specificity; operationally feasible
	[11]	Gazelle field evaluation	Magneto-optical scattering	Field	User-friendly; moderate sensitivity in low-density infections
RMOD (Rotating-Field Magneto-Optics)	[12]	Clinical RMOD detection for *P. vivax*	Rotating-field MO detection	Clinic/research	High detection for *P. vivax*; strong correlation with parasite density; LOD ~5 parasites/µL
	[13]	Large-scale RMOD evaluation (*n* = 956)	Rotating magnetic field → MO signal	Clinic	Sensitivity 82%; specificity 84%; better for *P. vivax*; detects residual hemozoin in recent infections
	[14]	Stage- & crystal-dependent RMOD physicochemical analysis	Frequency-dependent MO response	Laboratory	RMOD detects hemozoin amount + crystal-size distribution; explains stage-dependent variation
Magnetophoretic Lab-on-Chip	[15]	TMek chip capturing magnetized iRBC	Magnetic capture + impedance readout	Prototype/lab	High analytical sensitivity in early-phase prototype
Photoacoustic (PAFC/Cytophone)	[16]	‘Rainbow’ portable Cytophone using laser diodes	Dual-wavelength (808/915 nm) PAFC with time-color-coded signals	Preclinical (murine + in vitro *P. falciparum*)	Demonstrated portable PAFC platform; discriminates iRBCs, hemozoin, clots, and artefacts; detects iRBCs within hours of invasion with very low analytical LOD
	[17]	Noninvasive in vivo PA detection of malaria in Cameroon	1064-nm laser + focused ultrasound array; PA peaks from Hz in iRBCs	Clinical, hospital in an endemic setting	Cross-sectional + longitudinal trial (n = 20, 94 visits); sensitivity 90% and specificity 69% vs. microscopy; ROC–AUC 0.84; performance comparable to qPCR/RDT; safe and feasible noninvasive field prototype
Raman/SERS	[18]	SnSe_1.75_ nanoflake–Au SERS substrate for malaria detection	Defect-engineered semiconductor + SERS	Laboratory	LOD <100 infected RBC/mL; 100% detection in clinical validation; rapid (15 min) high-uniformity signal
	[19]	UTLC-SERS β-hematin quantification	Surface-enhanced Raman scattering	Laboratory	High analytical resolution; detects very low β-hematin concentrations
	[20]	2D-COS Raman mapping of hemozoin	Raman spectral correlation analysis	Laboratory	Captures structural transitions and hemozoin growth signatures
Optical/Spectrophotometric	[21]	UV–Vis–NIR spectral profiling of malaria stages	Optical absorption spectra	Laboratory	Stage-dependent absorbance is useful for differentiation
	[22]	Hyperspectral confocal imaging	Hyperspectral reflectance	Laboratory	Precisely differentiates trophozoite vs. schizont stages
	[23]	ON–OFF magneto-optical modulation spectroscopy	Field-modulated optical anisotropy	Laboratory	Stable spectrophotometric MO signal; not a diagnostic platform
NMR Relaxometry	[24]	T1/T2 relaxometry of infected RBCs	Magnetic relaxation perturbation by hemozoin	Laboratory	Distinct relaxation signatures; linked to maturation and pigment load
Smartphone-based Microscopy	[25]	Polarized smartphone microscope	Hemozoin birefringence	Clinic/prototype	Low-cost hemozoin imaging with high contrast
	[26]	WBC pigment visualization	Visualization of hemozoin granules	Hospital	Helps explain false-negative RDTs due to pigment sequestration
	[27]	Pigment-in-leukocyte scoring	Phagocytosed hemozoin quantification	Hospital	Predicts severity and mortality in severe malaria
Flow Cytometry	[28]	iPSC-RBC optical scatter detection	Hemozoin-induced light scatter	Research	Enables label-free detection of infected RBCs
Chemical Assay	[29]	Hemozoin-catalyzed ATRP polymerization	Radical polymerization catalysis	Laboratory	Ultra-low LOD; multi-step workflow
Nonlinear Optical Physics	[30]	Z-scan nonlinear optical hemozoin detection	Nonlinear optical absorption	Laboratory	Sensitive detection of nonlinear β-hematin response

### 3.3. Findings by Technology Group

#### 3.3.1. Magneto-Optical Detection

##### Static-Field Magneto-Optical Detection (Hz-MOD/Gazelle)

Five studies evaluated static-field MO technologies. De Melo (2021) and Fernando (2022) demonstrated robust specificity for *P. vivax* and *P. falciparum*, respectively, although sensitivity was reduced at low densities and early ring-stage infections [7,8]. Tan (2023) provided the first MO-based detection data for *P. knowlesi* [9]. Fontecha (2022) demonstrated strong operational feasibility in field settings [11], while Nallapati (2025) showed reproducibility across multiple sites [10]. Overall, static-field MO devices exhibited the highest technology readiness but continued to show limitations in detecting low-parasitic-density infections.

##### Rotating-Field Magneto-Optical Detection (RMOD)

**Figure 3:**
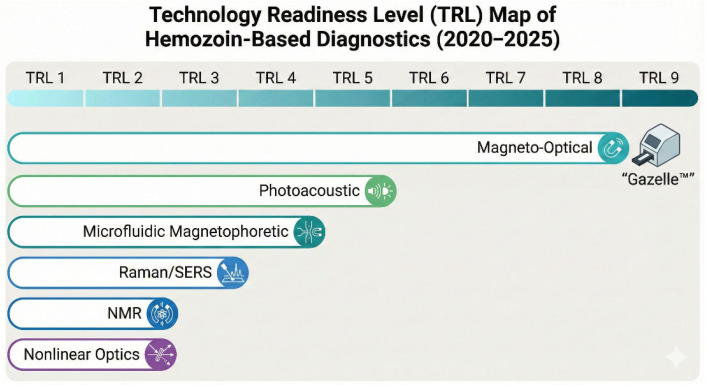
Technology Readiness Level (TRL) map of hemozoin-based malaria diagnostics (2020–2025). The figure illustrates the developmental status of various diagnostic technologies leveraging hemozoin. The horizontal scale indicates progression from TRL 1 (basic principles) to TRL 9 (system proven in operational environment). Colored bars and icons categorize the methods into magnetic (teal), acoustic (green), spectroscopic (blue), and optical (purple) families. Magneto-optical detection demonstrates the highest maturity (TRL 8–9), exemplified by the Gazelle™ device, while other methods remain in earlier research and development phases (TRL 1–5). TRL ranges represent variability reported across studies rather than a single averaged or best-case estimate.

Three studies examined rotating-field MO platforms. Orbán et al. (2021) demonstrated clear signal separation between *P. vivax* infections and uninfected controls, with MO amplitude scaling with parasitemia and detection thresholds approaching ~5 parasites/µL in laboratory conditions [12]. In a cross-sectional clinical study of 956 participants, Arndt et al. (2021) reported 82% sensitivity and 84% specificity for RMOD, with superior performance for *P. vivax* than *P. falciparum* [13]. Elevated RMOD signals in microscopy-negative individuals were attributed to persistence of circulating hemozoin following recent infections. Mechanistic analysis by Orbán et al. (2024) showed frequency-dependent MO responses that were dependent on hemozoin concentration and crystal size distribution, with schizont-derived crystals yielding higher signal amplitudes [14]. Overall, magneto-optical technologies remain the most mature diagnostic class, with TRL estimates ranging from 6 to 9 across platforms (Figure 3). Still, sensitivity for ring-stage *P. falciparum* remains limited, limiting early-infection detection.

#### 3.3.2. Magnetophoretic Lab-on-Chip (TMek Microdevice)

Giacometti et al. (2021) developed the TMek lab-on-chip [15], which incorporates high-gradient magnetic capture and impedance measurement to selectively retain hemozoin-containing infected erythrocytes in continuous flow. The device demonstrated clear impedance signatures that differentiated infected from uninfected cells, required low sample volumes (<10 µL), and maintained performance across a range of flow rates and parasitemia levels [15]. The TMek platform remains in the prototype stage but represents an emerging compact, reagent-free diagnostic pathway based on magnetophoretic enrichment coupled to electrical biosensing. The TMek microdevice demonstrates promising analytical sensitivity and minimal sample requirements, but remains at an early prototype stage (TRL 3–4) without clinical validation.

#### 3.3.3. Photoacoustic Detection (Cytophone/PAFC)

Jawad et al. (2022) developed a portable Cytophone using dual-wavelength (808/915 nm) laser diodes and time-color-coded photoacoustic peak analysis [16]. Using *P. falciparum* cultures and murine models, the system achieved ultralow analytical detection limits and distinguished infected erythrocytes from free hemozoin and clot artefacts. Yadem et al. (2024) performed the first noninvasive, in vivo clinical evaluation using a 1064-nm laser and a focused ultrasound detector to interrogate the dorsal hand veins of adults with uncomplicated malaria in Cameroon [17]. The device achieved 90% sensitivity, 69% specificity, and an ROC/AUC of 0.84. Sequential measurements showed a decline in the PA signal, consistent with qPCR-based parasite clearance. The study confirmed the feasibility of fully noninvasive hemozoin-based diagnosis. These findings position photoacoustic Cytophone systems as the only hemozoin-based technology with demonstrated noninvasive in vivo performance, although current devices remain precommercial (TRL 4–5).

#### 3.3.4. Raman and SERS-Based Diagnostics

Xu et al. (2022) fabricated SnSe_1.75_ nanoflake-Au heterostructures with engineered selenium vacancies, producing synergistic near- and far-field SERS enhancement [18]. The platform detected infected RBCs at <100 cells/mL and achieved full diagnostic concordance across 160 measurement points from clinical blood samples. Yadav et al. (2023) applied UTLC-SERS to quantify β-hematin at high spectral resolution, demonstrating excellent analytical performance on laboratory standards [19]. Migdalski et al. (2025) employed 2D-correlation Raman spectroscopy to characterize molecular transitions during hemozoin crystal growth, providing insights into the developmental dynamics of the parasite [20]. While analytically powerful, all SERS methods require specialized substrates and instrumentation, limiting immediate translation.

#### 3.3.5. Optical, Spectrophotometric, and Hyperspectral Imaging

Baptista et al. (2023) characterized UV-Vis-NIR spectra of infected blood, identifying stage-dependent absorbance features useful for differentiating trophozoites and schizonts [21]. Kwon et al. (2025) applied hyperspectral confocal imaging to classify parasite developmental stages with high discrimination accuracy [22]. Kara et al. (2021) introduced ON-OFF magneto-optical modulation spectroscopy to characterize anisotropic optical responses of β-hematin crystals [23]. Although primarily physicochemical in scope, this work provides foundational parameters for future optical detection strategies. These techniques offer strong mechanistic and staging insights, but translation into deployable diagnostics is limited by the complexity of the hardware (TRL 2–3).

#### 3.3.6. NMR Relaxometry

Di Gregorio et al. (2020) demonstrated that suspensions of *P. falciparum*-infected erythrocytes exhibited progressive shortening of T2 and characteristic perturbations to T1 relaxation as hemozoin content increased during parasite maturation [24]. Ring-stage parasites generated minimal changes, whereas trophozoite- and schizont-rich samples caused strong relaxation alterations consistent with superparamagnetic crystal effects. While mechanistically informative, the requirement for high-field NMR instruments restricts translation beyond laboratory settings. NMR relaxometry provides a mechanistic insight into pigment accumulation but is not yet suitable for diagnostic deployment (TRL 1–2).

#### 3.3.7. Smartphone Microscopy and Pigment-Containing Leukocytes

Yu et al. (2023) demonstrated that polarized smartphone microscopy can visualize hemozoin birefringence with good contrast, offering a low-cost platform for laboratory detection [25]. Two studies focused on pigment-containing leukocytes. Eeckhout et al. (2021) described how hemozoin-laden neutrophils and monocytes may contribute to false-negative RDT results [26], while Srinamon et al. (2022) found strong associations between leukocyte pigment burden and clinical severity [27]. These markers are more prognostic than diagnostic but highlight the broader relevance of hemozoin detection in host immune cells. Smartphone-based imaging presents a feasible, low-cost pathway for field use (potential TRL 5–6), although pigment-in-leukocyte studies are more prognostic than diagnostic.

#### 3.3.8. Flow Cytometry

Pance et al. (2023) evaluated a label-free detection strategy using induced pluripotent stem cell-derived erythrocytes infected with *P. falciparum* [28]. The study demonstrated that hemozoin crystals within infected erythrocytes generate distinctive forward- and side-scatter signatures due to their size, density, and refractive index contrasts relative to uninfected cells. These scatter profiles enabled the differentiation of infected cells without the need for fluorescent staining, thereby reducing workflow complexity. The authors also examined variability across parasite stages, noting that trophozoite- and schizont-stage parasites produced more pronounced scatter shifts, consistent with increased crystal accumulation. While promising, the method’s performance was evaluated solely in controlled in vitro systems, and no clinical blood samples were tested. Additionally, flow cytometry requires specialized benchtop instrumentation and trained operators, which limits its immediate field applicability. The technology remains of interest as a high-throughput analytical tool for hemozoin quantification or drug-susceptibility research, with potential future integration into miniaturized cytometry platforms. This approach remains limited by equipment requirements and has not yet been validated in clinical samples, placing it at TRL 2–3.

#### 3.3.9. Chemical and Nonlinear Optical Approaches

Raccio et al. (2020) used β-hematin to catalyze ATRP polymerization, enabling ultrasensitive pigment detection through colorimetric shifts, though requiring multi-step reactions [29]. Sahoo et al. (2024) used Z-scan nonlinear optics to characterize the absorption properties of β-hematin, providing foundational optical parameters [30]. These methods remain in the early-stages and primarily contribute physicochemical insight rather than deployable diagnostics (TRL 1–2).

Across the 24 included studies, magneto-optical platforms exhibited the highest readiness for field deployment, whereas photoacoustic systems demonstrated the first evidence of noninvasive in vivo detection. Raman/SERS approaches showed superior analytical sensitivity in laboratory settings, while microfluidic and smartphone-based platforms offer emerging low-resource solutions. Collectively, the findings indicate substantial technological diversity but uneven clinical validation across platforms, highlighting the need for standardized benchmarking and comparative evaluation.

Table 1 summarizes 24 studies evaluating hemozoin-based diagnostic, analytical, and preclinical technologies for malaria detection. Studies are grouped into major technology categories, including magneto-optical platforms (e.g., Gazelle, Hz-MOD, RMOD), photoacoustic systems (PAFC/Cytophone), Raman/SERS approaches, optical–spectrophotometric techniques, NMR relaxometry, smartphone-based microscopy, flow cytometry, and chemical assays. Each entry provides a concise description of methodological principles, detection physics, typical deployment settings, and the main findings reported by the authors. Across studies, magneto-optical platforms demonstrated the highest readiness for field use (TRL 7–9), whereas photoacoustic, Raman/SERS, and NMR approaches remain predominantly laboratory or preclinical tools (TRL 2–5). The table supports cross-comparison of methodological maturity, performance characteristics, and implementation feasibility.

## 4. Discussion

This scoping review synthesized findings from 24 studies assessing hemozoin-targeted malaria diagnostics. These studies span magneto-optical, photoacoustic, Raman/SERS, optical, microfluidic, and physicochemical modalities, reflecting rapid innovation in reagent-free diagnostic strategies. Against the backdrop of ongoing challenges posed by low-density infections and the limitations of microscopy and RDTs [31,32], the evidence highlights both the promise and current constraints of hemozoin-based detection.

### 4.1. Advances in Hemozoin-Targeted Diagnostic Technologies

Magneto-optical technologies constitute the most developed diagnostic class. RMOD platforms demonstrated strong correlations between MO amplitude and parasitemia [12], while large-scale clinical evaluation showed moderate to high accuracy, particularly for *P. vivax* [13]. Static-field devices such as Hz-MOD and Gazelle demonstrated high specificity and robust performance under field conditions [4,9,10,11], consistent with earlier reports that identified MO signals as reliable hemozoin markers [33].

Photoacoustic flow cytometry (Cytophone) represents a conceptually distinct innovation. Preclinical studies demonstrated ultralow analytical detection of infected erythrocytes [16], and the first noninvasive clinical evaluation showed strong sensitivity for *P. falciparum* infections [17]. These results align with the broader photoacoustic literature, which shows that hemozoin is an efficient broadband absorber suitable for in vivo detection [34,35].

Raman and SERS platforms demonstrated some of the highest analytical sensitivities. Defect-engineered SnSe_1.75_–Au substrates enabled highly reproducible detection below 100 iRBC/mL [18], consistent with earlier work showing that hemozoin exhibits sharp, distinctive vibrational peaks [36]. Although promising, these platforms rely on specialized instrumentation and remain primarily analytical.

Additional diagnostic modalities—including the TMek magnetophoretic lab-on-chip [15], smartphone-based polarized microscopy [25], flow cytometry [28], spectrophotometry [21], NMR relaxometry [24], and nonlinear optics [30]—provide complementary contributions across usability, throughput, and mechanistic insight.

### 4.2. Detection Performance and Biological Determinants

Direct cross-platform performance comparison is constrained by heterogeneous reporting units, biological matrices, sample preparation workflows, and reference standards, limiting the interpretability of absolute sensitivity claims. Across the reviewed studies, detection limits are variably reported in units such as parasites/µL, infected red blood cells per milliliter, or β-hematin equivalents, often without harmonized consideration of sample volume or matrix complexity. In addition, reported analytical performance frequently depends on upstream processing steps, including blood lysis, magnetic enrichment, microfluidic concentration, or substrate-specific signal amplification, which are not uniformly described or standardized. Clinical performance metrics such as sensitivity, specificity, and area under the curve are further influenced by differences in reference standards, transmission settings, and patient populations. As a result, apparent differences in reported performance across platforms may reflect experimental context and methodological design rather than intrinsic superiority of a given detection principle. These limitations underscore the need for standardized benchmarking frameworks to enable meaningful comparative evaluation of hemozoin-based diagnostic technologies.

Across technologies, detection performance reflected parasite biology. RMOD, Hz-MOD, and flow cytometry showed improved performance for trophozoite- and schizont-rich samples, consistent with the exponential accumulation of hemozoin across the erythrocytic cycle [37]. Sensitivity for early ring-stage infections remained limited—especially for *P. falciparum*—reflecting longstanding knowledge of minimal pigment content in early infections [38]. Reduced detectability of early ring-stage parasites reflects minimal hemozoin accumulation during early hemoglobin digestion, compounded by sequestration dynamics and low circulating pigment burden. Future sensor technologies may partially overcome this limitation through larger effective sampling volumes, signal integration strategies, or multimodal approaches that combine hemozoin detection with complementary biomarkers. Importantly, hemozoin persistence may yield detectable signals in parasitaemia-negative individuals following recent or resolved infection, thereby reducing specificity for active malaria. This limitation is not confined to a single detection principle, but is shared across magneto-optical, photoacoustic, and spectroscopic modalities, as all rely on the presence of the pigment rather than viable parasites. Consequently, modality-specific performance differences should be interpreted in the context of this shared biological constraint rather than attributed solely to technical sensitivity.

SERS-based platforms achieved high sensitivity under laboratory conditions, but their performance in unprocessed whole blood remains to be fully established. Photoacoustic Cytophone demonstrated strong clinical sensitivity, though modest specificity suggests overlap with other vascular absorbers—a phenomenon documented in earlier photoacoustic analyses [39,40]. Interpretation of this moderate specificity requires consideration of competing photoacoustic signals from endogenous vascular absorbers, including hemoglobin, as well as inter-individual anatomical and physiological variability. Measurement conditions, calibration procedures, and algorithmic classification strategies further influence signal discrimination and may contribute to false-positive classifications. Importantly, these factors suggest that current specificity limitations are more likely related to engineering, signal processing, and analytical thresholds than to an inherent constraint of the photoacoustic detection principle itself.

These trends are epidemiologically important, given the increasing recognition that submicroscopic and asymptomatic infections form a substantial reservoir of transmission [28] and often evade detection by conventional RDTs [41].

Across the reviewed platforms, limits of detection (LOD) varied substantially by detection principle and biological context (Supplementary Table S1). Magneto-optical systems typically achieved detection thresholds in the range of ~5–100 parasites/µL under laboratory or clinical conditions, with reduced sensitivity for early ring-stage *P. falciparum* [7,8,9,10,11]. Photoacoustic Cytophone platforms demonstrated ultralow analytical sensitivity and the capacity for in vivo detection, although clinical specificity varied [16,17]. Raman/SERS-based approaches achieved the lowest analytical LODs, in some cases below 100 infected cells/mL [18], but remain confined to laboratory settings. Emerging microfluidic and smartphone-based platforms aim to balance cost, robustness, and diagnostic accuracy by leveraging simplified workflows, low-cost components, and minimal infrastructure requirements. While these approaches often exhibit higher detection limits compared to laboratory-based systems, their reduced cost, portability, and ease of use may offer practical advantages in resource-limited malaria-endemic settings, particularly for screening and surveillance applications. The higher operational readiness of magneto-optical platforms reflects system-level advantages, including simpler hardware architectures, lower power requirements, shorter assay times, and demonstrated robustness in field evaluations, rather than superior analytical sensitivity alone [1,12,13,33]. In contrast, Raman/SERS and photoacoustic systems often require more complex instrumentation, calibration, and signal processing, which currently limit their deployability despite promising analytical performance [17,21,34,36].

At low parasitemia levels typical of asymptomatic infections, detection performance across hemozoin-based platforms diverges substantially [32,41,42]. Magneto-optical and photoacoustic approaches generally retain higher sensitivity due to their ability to integrate signals across larger sample volumes or in vivo detection [1,12,17], whereas Raman/SERS-based methods achieve high analytical sensitivity under controlled conditions but are more susceptible to matrix effects in whole blood [12,21]. Across platforms, specificity at low parasite densities is further influenced by biological factors such as hemozoin persistence and background absorbers [27,38,43].

### 4.3. Readiness for Implementation in Malaria-Endemic Settings

Technology readiness varied widely (Supplementary Table S1). Static-field MO systems appear ready for field deployment, supported by multi-country evaluations and operational robustness even in resource-limited environments [8,11]. These characteristics align with the WHO diagnostic criteria, emphasizing stability, a low training burden, and a rapid turnaround.

RMOD tools provide additional insights into the hemozoin crystal size distribution [14], although current devices are less portable. The TMek microdevice [15] illustrates a promising route toward miniaturized diagnostics through magnetic enrichment coupled with electrical readouts, a trend observed in the development of microfluidic biosensor [44].

Cytophone devices demonstrate a radically different paradigm by enabling noninvasive, in vivo detection, but require further engineering refinement before widespread implementation. Raman/SERS, hyperspectral imaging, NMR relaxometry, and nonlinear optics remain laboratory-focused, contributing primarily mechanistic or analytical insight.

An inherent limitation shared by all hemozoin-based diagnostic approaches is the biological persistence of hemozoin following parasite clearance. Hemozoin can remain detectable within circulating erythrocytes, phagocytic leukocytes, and tissue-resident macrophages for prolonged periods after effective antimalarial treatment [45,46,47]. In high-transmission settings, this persistence may complicate the interpretation of diagnostic signals by reducing specificity for active infection, particularly when low-density parasitemia is targeted. This challenge parallels a well-recognized limitation of HRP2/3-based rapid diagnostic tests, where antigen persistence can lead to false-positive results [48,49].

Importantly, hemozoin persistence represents a biological constraint rather than a platform-specific limitation and should therefore be considered during clinical validation, benchmarking, and the interpretation of analytical sensitivity across detection modalities.

### 4.4. Integration with Prognostic Markers and Host Response

Microscopy-based studies in this review supported the broader literature, which identifies pigment-containing leukocytes (HCL) as clinically meaningful markers. Earlier studies established that intraleukocytic hemozoin correlates with disease severity and mortality [43]. Subsequent pooled analyses reinforced its prognostic value across African and Asian populations.

Findings from Eeckhout et al. (2021) and Srinamon et al. (2022) complement this evidence, highlighting the diagnostic implications of pigment-laden leukocytes for RDT performance and their strong association with severe malaria phenotypes [26,27]. Together, these data suggest that hemozoin-targeted technologies could be expanded beyond parasite detection to monitor cumulative parasite burden and inflammatory risk.

### 4.5. Evidence Gaps and Research Priorities

Key gaps identified include:

- Limited evaluation in low-density and asymptomatic infections, despite their epidemiological significance.
- Small, geographically narrow clinical studies limit generalizability.
- Incomplete species coverage, with data lacking for *P. knowlesi*, *P. malariae*, and *P. ovale*.
- Lack of head-to-head comparisons across magneto-optical, photoacoustic, SERS, and optical modalities.
- Sparse pediatric data, despite a high malaria burden in children.

Addressing these gaps will require multicenter studies with standardized molecular reference tests and assessment of post-treatment pigment persistence [50].

### 4.6. Overall Interpretation

Hemozoin-based technologies form a diverse and rapidly maturing diagnostic landscape. Magneto-optical devices are nearing operational deployment; photoacoustic approaches introduce the first clinically feasible noninvasive detection pathway; and Raman/SERS platforms define the upper limits of analytical sensitivity. These developments align with broader scientific understanding of hemozoin’s unique magnetic and optical signatures [36] and highlight its continued relevance as a reagent-free biomarker for malaria diagnosis. Future progress will depend on rigorous clinical validation, improved low-density performance, and engineering pathways aligned with practical constraints in endemic settings.

### 4.7. Relevance to the Sustainable Development Goals (SDGs)

This scoping review aligns directly with the Sustainable Development Goals, particularly SDG 3 (Good Health and Well-Being), and the malaria elimination targets embedded within it. By mapping emerging hemozoin-based technologies that aim to provide rapid, reagent-free, and potentially noninvasive diagnostics suitable for use in low-resource settings, the review highlights the need for more sensitive tools to detect low-density and asymptomatic infections, which are increasingly recognized as key obstacles to malaria control and elimination in endemic regions. In addition, innovations such as portable magneto-optical devices, microfluidic lab-on-chip platforms, smartphone-based microscopy, and noninvasive photoacoustic Cytophone systems contribute to SDG 9 (Industry, Innovation and Infrastructure) and support more equitable access to high-quality diagnostics in low- and middle-income countries, thereby indirectly advancing SDG 1 (No Poverty) and SDG 10 (Reduced Inequalities) by helping to reduce the health and economic burden of malaria among the most vulnerable populations.

### 4.8. Implications for Practice

Hemozoin-targeted diagnostics offer several practical opportunities for malaria-endemic settings. Magneto-optical platforms—particularly Gazelle™ and similar devices—represent the closest near-term alternative to antigen-based rapid diagnostic tests, especially in regions with pfhrp2/pfhrp3 deletions or high rates of submicroscopic infection. Photoacoustic Cytophone systems introduce a novel noninvasive diagnostic paradigm, although they require further engineering adaptation before field deployment. Smartphone-based microscopy and microfluidic magnetophoretic devices offer promising, low-cost pathways that align with the operational needs of primary care facilities in low-resource regions. These technologies may complement existing diagnostic algorithms by improving the detection of low-density infections and offering reagent-free workflows.

### 4.9. Implications for Research

Future research should prioritize head-to-head comparative studies under standardized conditions to enable meaningful benchmarking across magneto-optical, photoacoustic, Raman/SERS, and microfluidic platforms. Larger multicenter clinical evaluations are needed to assess performance across diverse epidemiological contexts, including low-density asymptomatic infections and infections caused by non-Falciparum species. Engineering research should focus on reducing hardware complexity, improving ring-stage sensitivity, and integrating quality-control mechanisms suitable for field use. Economic and operational evaluations—including cost-effectiveness, device durability, and workflow integration—are essential for guiding policy adoption in endemic settings.

The findings highlight both the scientific promise and operational challenges of hemozoin-based diagnostics. While magneto-optical platforms are nearing practical deployment, next-generation technologies such as photoacoustics, Raman/SERS, and microfluidics continue to expand the diagnostic frontier. Closing the remaining evidence gaps—especially in low-density infections, species diversity, and field performance—will be crucial to advancing these platforms from proof of concept toward routine use in malaria-endemic settings.

Across the reviewed literature, several recurring sources of bias and technical limitation emerge that collectively shape the interpretability and translational relevance of hemozoin-based diagnostic technologies. Biological determinants, including parasite developmental stage and post-treatment persistence of hemozoin, directly influence detectable signal strength and diagnostic specificity. Sample-related factors such as blood volume, matrix complexity, and preparation workflows further contribute to variability in reported performance across platforms. In parallel, technical and algorithmic dependencies—including signal processing strategies, classification thresholds, and calibration approaches—introduce additional sources of bias that are often underreported. Finally, the absence of standardized benchmarking frameworks and harmonized reporting metrics complicates cross-platform comparison and may obscure true performance differences. Together, these factors underscore the importance of integrated biological, technical, and methodological considerations when interpreting current evidence and designing future validation studies.

Meaningful cross-platform comparison and regulatory progress will require standardized benchmarking frameworks, including harmonized reporting units, well-characterized reference materials such as synthetic hemozoin or standardized infected blood panels, and multicenter clinical validation studies across diverse transmission settings. Alignment with consensus performance metrics and established regulatory pathways will be essential to enable objective comparison and accelerate the translation of hemozoin-targeted diagnostics into routine clinical and public health use.

## 5. Conclusions

This scoping review identified 24 studies describing a broad spectrum of hemozoin-based malaria detection technologies, ranging from magneto-optical and photoacoustic systems to Raman/SERS platforms, microfluidic magnetophoretic devices, optical and hyperspectral imaging, NMR relaxometry, and cellular imaging approaches. These technologies leverage diverse physical principles—including magnetic anisotropy, optical absorption, vibrational signatures, and nonlinear optical responses—to detect hemozoin as a reagent-free biomarker of malaria infection.

Across platforms, magneto-optical devices emerged as the most operationally advanced, demonstrating robust specificity and field feasibility, while photoacoustic Cytophone systems provided the first evidence of noninvasive in vivo detection. Raman/SERS platforms achieved the highest analytical sensitivity, whereas TMek magnetophoretic chips, smartphone microscopy, and flow cytometry offered intermediate-complexity routes with potential for miniaturization. Nonetheless, important limitations remain, including reduced sensitivity for early ring-stage *P. falciparum*, limited validation in low-density and asymptomatic infections, and incomplete species coverage.

Overall, this review demonstrates that hemozoin-based diagnostics constitute a rapidly maturing and methodologically diverse field. Magneto-optical platforms are closest to operational deployment, while photoacoustic approaches introduce the first clinically validated noninvasive diagnostic pathway. Raman/SERS technologies define the upper limits of analytical sensitivity but remain laboratory-bound. Future research should prioritize standardized head-to-head evaluations, large-scale clinical validation, and improved detection of low-density infections to support the translation of hemozoin-based diagnostics into routine use in malaria-endemic, resource-limited settings.

## Data Availability

All data supporting the findings of this study are available within the article and its Supplementary Materials.

## Supplementary Materials

The following supporting information can be downloaded at: https://www.mdpi.com/article/10.3390/bios16010048/s1, Table S1: Characteristics of Included Studies on Pigment-Targeted Malaria Detection Technologies (*n* = 24).

## Abbreviations

The following abbreviations are used in this manuscript:

ATRP	Atom Transfer Radical Polymerization
COS	Two-Dimensional Correlation Spectroscopy
Cytophone	Photoacoustic flow cytometry system
Hz	Hemozoin
Hz-MOD	Hemozoin Magneto-Optical Detection
iRBC	Infected red blood cells
iPSC-RBC	Induced pluripotent stem cell-derived red blood cells
LOD	Limit of detection
ML	Machine learning
MO	Magneto-optical
NIR	Near-infrared
NMR	Nuclear magnetic resonance
PA	Photoacoustic
PAFC	Photoacoustic flow cytometry
RMOD	Rotating Magneto-Optical Detection
SERS	Surface-Enhanced Raman Scattering
SPOI	Smartphone polarized optical imaging
T1/T2	Longitudinal and transverse relaxation times
TRL	Technology Readiness level
WBC	White Blood Cells
